# Supplementary material for: Genome-wide screens reveal shared and strain-specific genes that facilitate enteric colonization by Klebsiella pneumoniae
Source: mBio. 2023 Oct 25;14(6):e02128-23. doi: 10.1128/mbio.02128-23 (PMC10746194; doi:10.1128/mbio.02128-23)
Supplement: Supplemental Figures — Figures S1 to S7. [file mbio.02128-23-s0005.pdf]

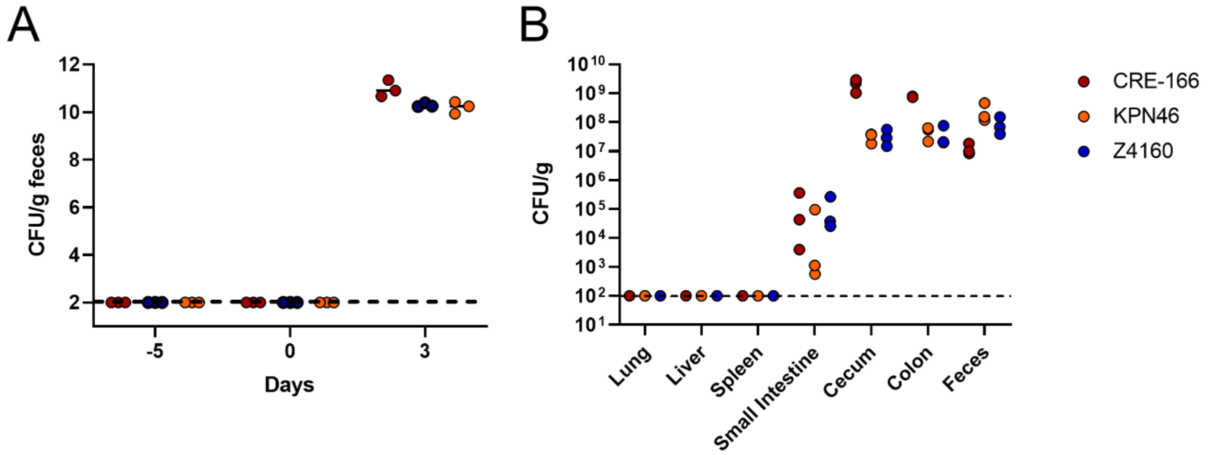

Supplemental Figure 1 Accuracy of detection of *K. pneumoniae* in a gut-limited model of colonization. (A) CFU recovered from mouse feces collected before experimental intervention (Day -5), after daily vancomycin injections (Day 0), and after inoculation with transposon mutant libraries (Day 3). Feces were homogenized and plated on LB agar supplemented with carbenicillin. (B) CFU recovered from organs collected at Day 14 post-gavage with *K. pneumoniae*. Organs were homogenized and plated on LB agar supplemented with carbenicillin.

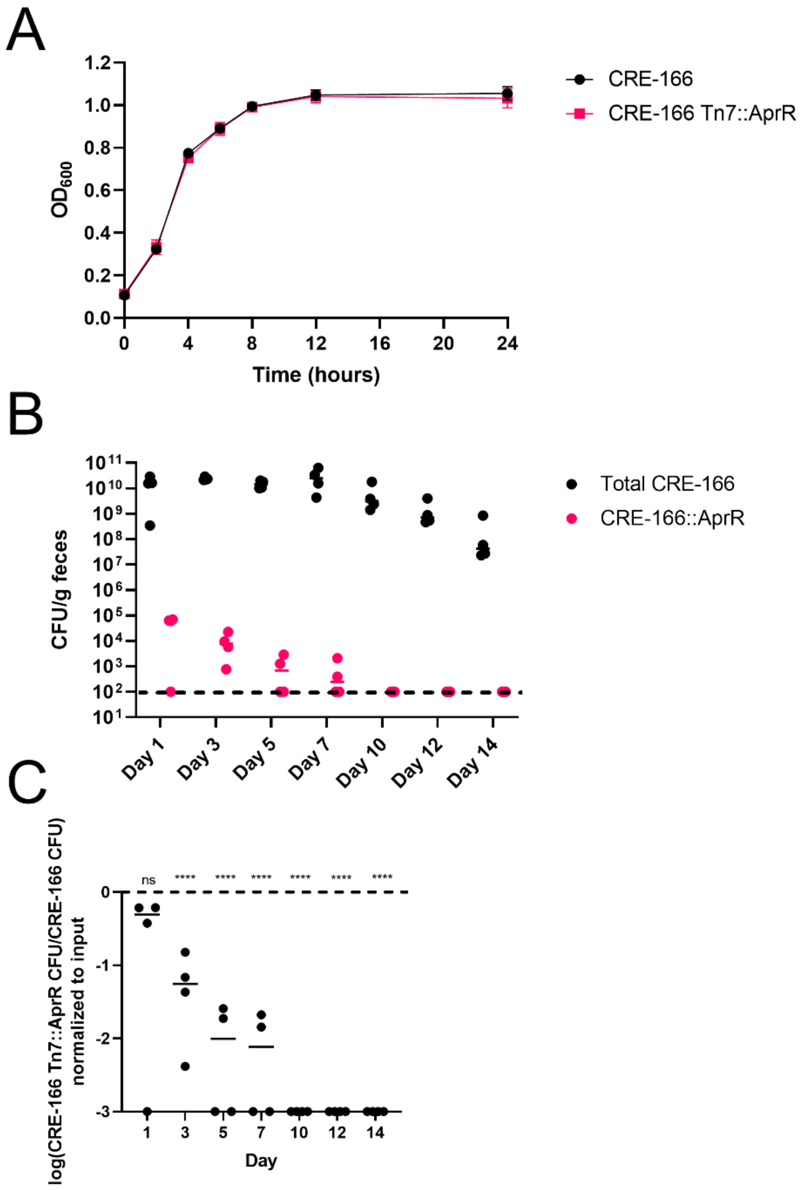

Supplemental Figure 2 Validation and use of a marked *K. pneumoniae* strain to examine bottlenecks in the mouse model of GI colonization. (A) Growth curve in LB for parent strain, CRE-166, and marked strain CRE-166 Tn7::AprR. Points indicate an average of 3 technical replicates with a standard deviation marked in error bars. This is a representative curve from 3 biological replicates. (B) *In vivo* bottleneck detection experiment performed by spiking a marked strain, CRE-166 Tn7::AprR, into an inoculum of CRE-166 at a ratio of 1:100,000. Fecal samples were collected and CFU enumerated. (C) Competitive indices calculated for data displayed in panel B. Asterisks denote significance by one-sample t-tests with Dunn correction where **** $p < 0.0001$, and “ns” indicates not significant.

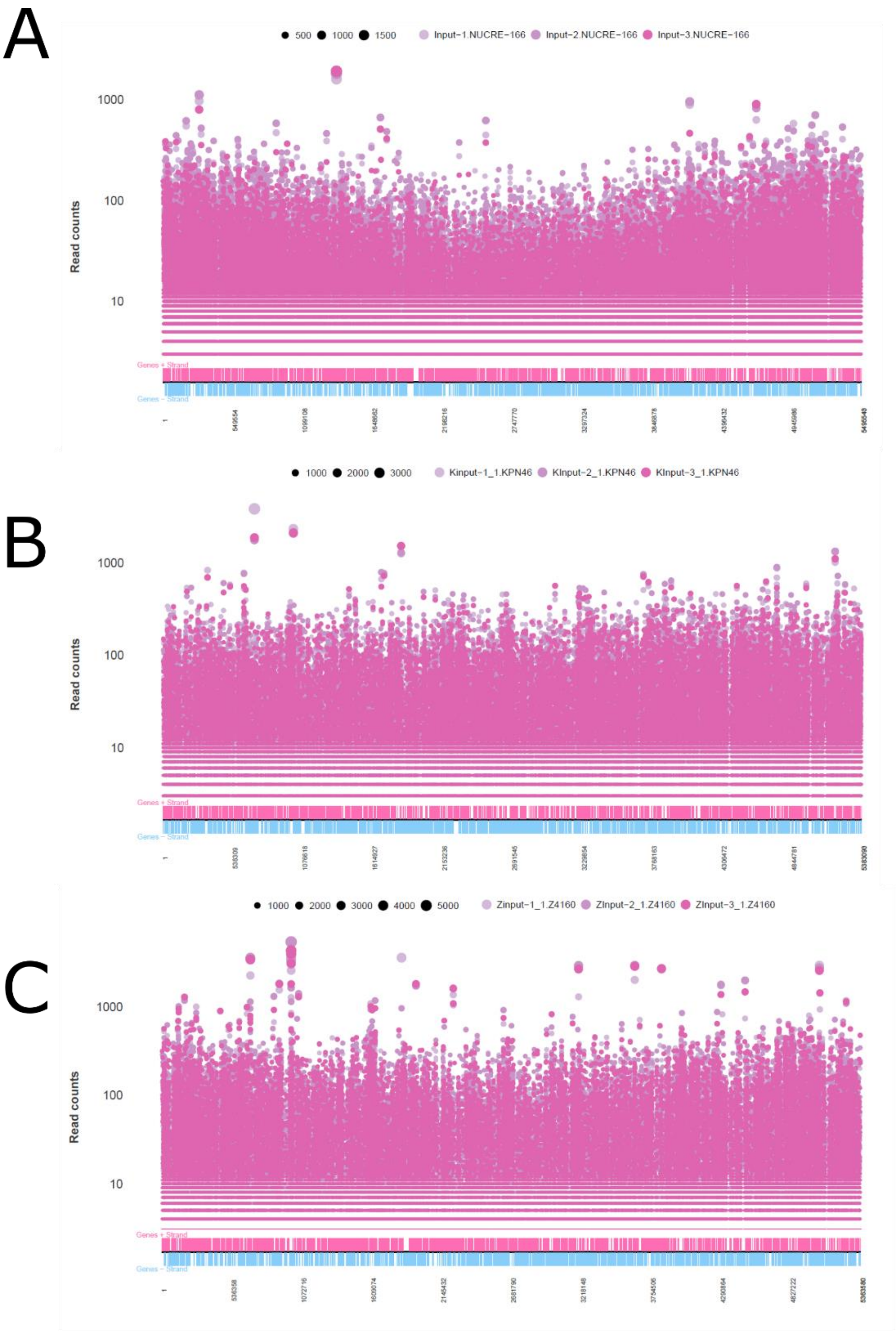

Supplemental Figure 3 Distribution of transposon insertion sites and sequence read numbers across the *K. pneumoniae* chromosome. Insertion sites and read numbers are shown for (A) CRE-166, (B) KPN46, and (C) Z4160. Pink dots indicate reads of insertion sites with both the y-axis and size of the dot indicating number of reads. The first track below denotes CDS on the positive strand, and the lowest track indicates CDS on the negative strand.

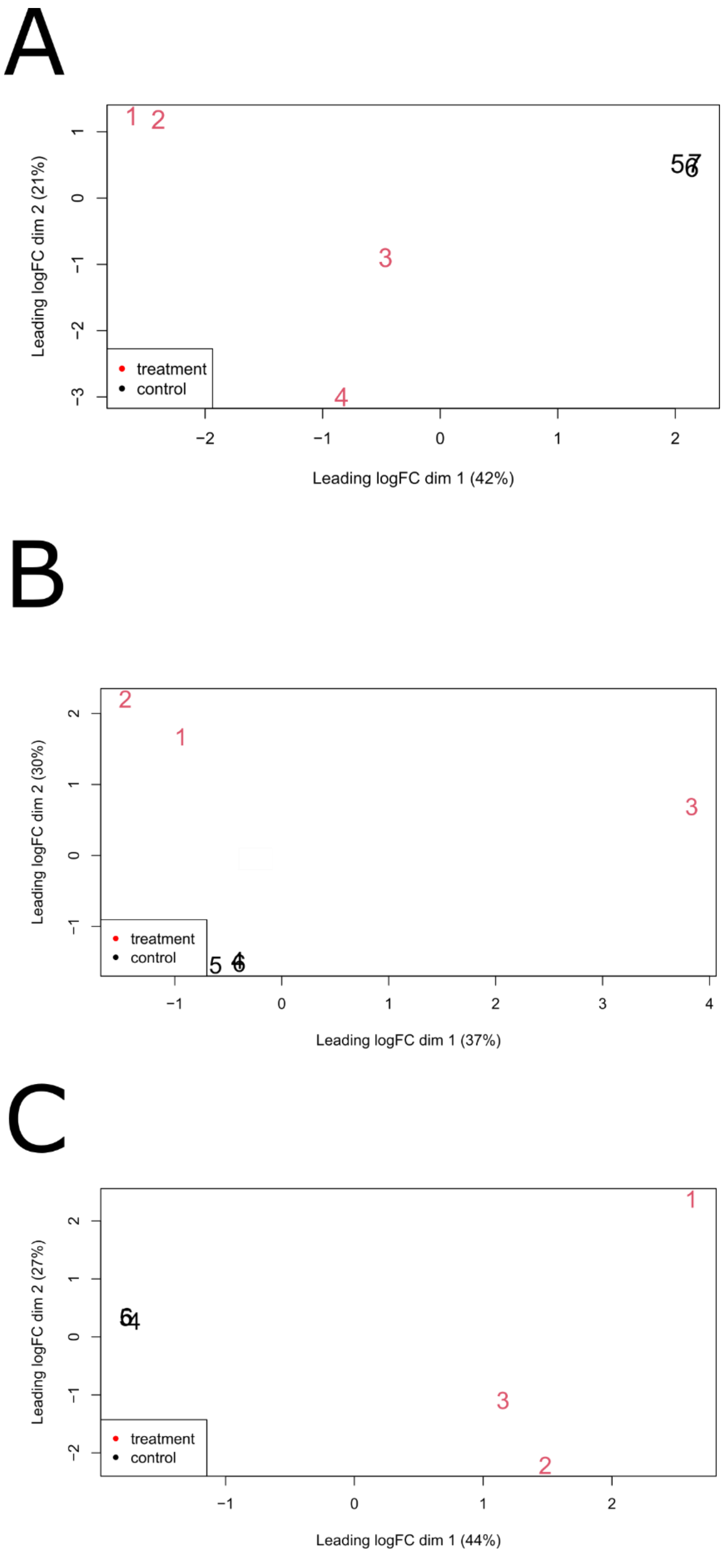

Supplemental Figure 4 Multidimensional scaling (MDS) plots for transposon insertion sequencing results. Input control pools (black font) and outputs from the treatment (GI colonization) (red font) for transposon insertion mutant screens in (A) CRE-166, (B) KPN46, and (C) Z4160 are indicated.

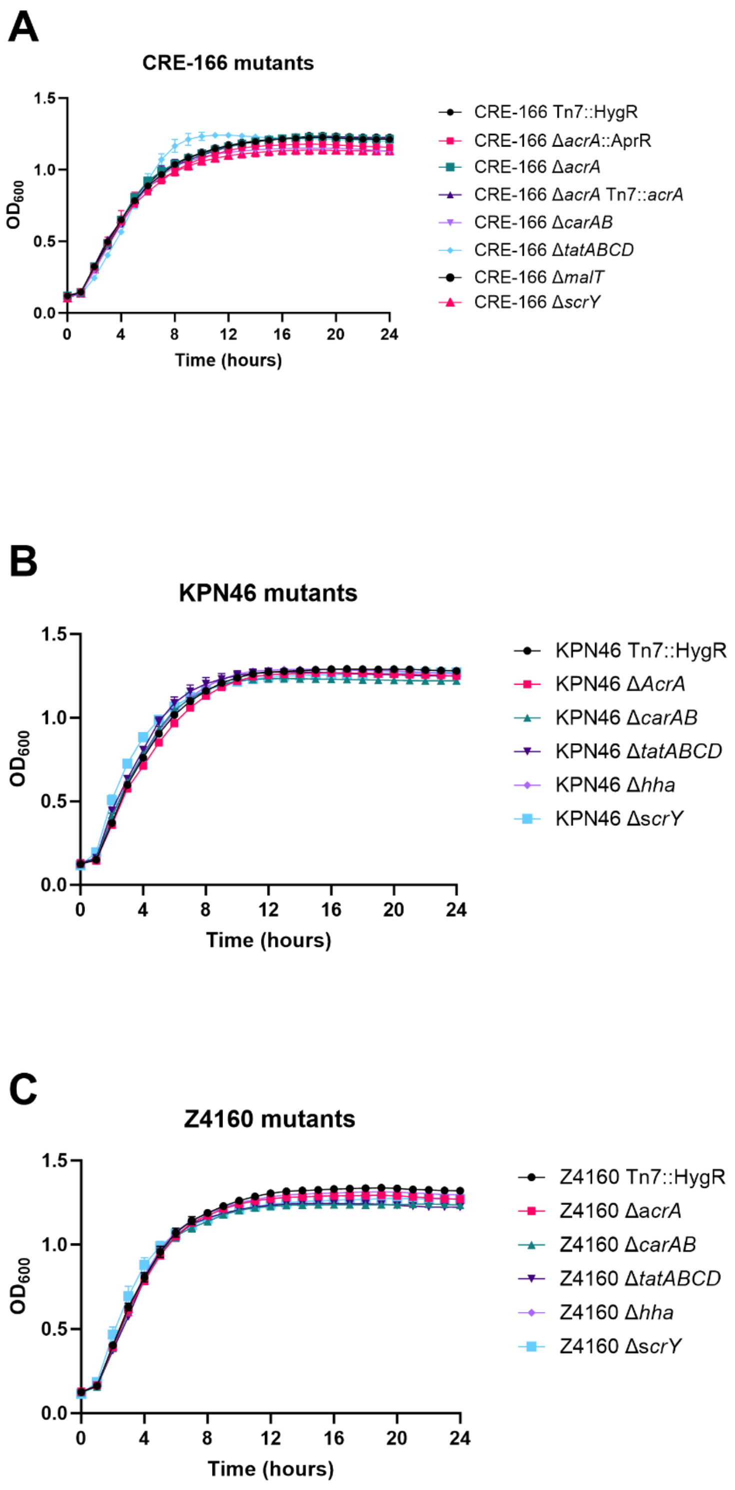

Supplemental Figure 5 Growth curves in LB for marked parental strains and isogenic mutants of *K. pneumoniae*. Growth curves are for (A) CRE-166, (B) KPN46, and (C) Z4160.

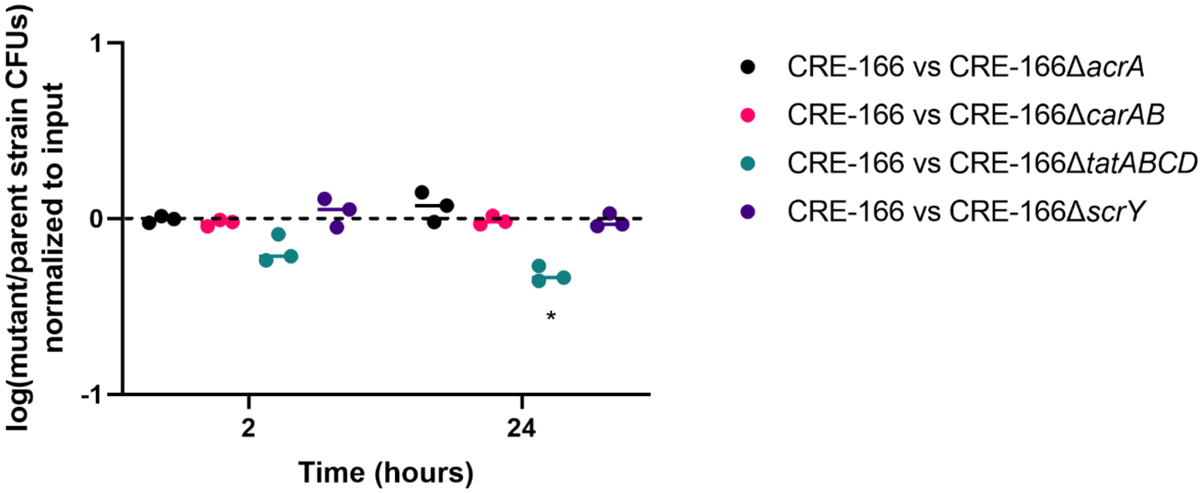

Supplemental Figure 6 *In vitro* competition experiments between marked parental strains and isogenic mutants of *K. pneumoniae*. Strains were inoculated in a 1:1 mixture into LB, incubated, and CFU were plated for enumeration at the indicated timepoints. n = 3 biological replicates. Line denotes median. * indicates $p < 0.05$ in one-sample t-tests with Dunn correction. Log(competitive index) = 0, or equal recovered CFU of parental strain and mutant, is marked with a dashed line.

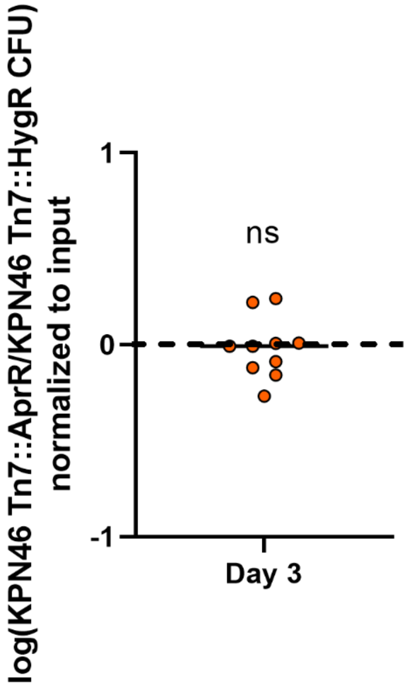

Supplemental Figure 7 Impact of off-site nonsynonymous single nucleotide variants found in the *carAB* mutants on GI colonization. Competitive colonization experiments between two marked parental KPN46 strains, one (Apr^R) with the nucleotide variants and one (Hyg^R) without them, were performed. Mice were treated with 5 days of vancomycin prior to gavage with 1:1 mixtures of two parental strains marked with either a hygromycin-resistance cassette or an apramycin-resistance cassette at the Tn7 site. Bacterial CFU recovered from the feces at Day 3 were quantified. n = 10. “ns” indicates not significant one-sample t-tests with Dunn correction. Log(competitive index) = 0, or equal recovered CFU of both marked parental strains, is indicated with a dashed line.
