# Supplementary material for: Genome-wide screens reveal shared and strain-specific genes that facilitate enteric colonization by Klebsiella pneumoniae
Source: mBio. 2023 Oct 25;14(6):e02128-23. doi: 10.1128/mbio.02128-23 (PMC10746194; doi:10.1128/mbio.02128-23)
Supplement: Supplemental text — Supplemental methods. [file mbio.02128-23-s0006.docx]

**Supplemental Methods**

Preparation of Complete Genomes

Genomic DNA was purified using the Maxwell 16 system (Promega). Short-read genome assemblies for CRE-166 (accession number: SAMN17600204) and KPN46 (accession number: SAMN24040734) were published previously^1,2^. For Z4160, a Nextera XT kit (Illumina) was used for library preparation before sequencing on an Illumina MiSeq. Sequences were trimmed using Trimmomatic v0.32 ^3^, and *de novo* assembly was performed with SPAdes 3.9.1. Contigs were removed if they were shorter than 200 bp or had a mean fold coverage of <5x per base. Long-read Nanopore sequencing libraries for CRE-166, KPN46, and Z4160 were prepared using the ligation sequencing kit (SQK-LSK109, Oxford Nanopore) and sequenced on a MinION using a FLO-MIN106 flow cell. Base calling and demultiplexing of sequence reads was performed using Guppy v3.4.5^4^. Hybrid assembly and circularization of Nanopore and Illumina reads were performed using Flye v2.9. Nanopore sequencing errors were corrected by aligning Illumina reads to the assembly using BWA v0.7.17 followed by serial rounds of Pilon v1.23.

Construction of pSAM*hygSDM*

The erythromycin-resistance cassette of pSAM*erm* was replaced with a hygromycin-resistance cassette (HygR) amplified from the pFLP-hyg plasmid (Addgene plasmid #87831^5^) with primers KEB306 and 307 (which also added MfeI and XbaI sites, respectively) and ligated into the cloning vector pCR®2.1-TOPO (Invitrogen). (All primers used in this study are listed in Supplemental Table 3.) The hygromycin-resistance cassette contained two internal MmeI sites, which had the potential to interfere with amplification of transposon-flanking DNA by the Goodman technique^6^. Thus, these sites were removed as follows. The Phusion Site-Directed Mutagenesis Kit (Thermo Scientific) was used iteratively to perform site-directed mutagenesis twice (round 1: primers KEB308, 309; round 2: KEB310, 311) to introduce synonymous changes at amino acids serine 23 and serine 201 (cytosine to adenosine nucleotide changes for both sites). These changes destroyed the internal MmeI recognition sites but maintained protein sequence, yielding a modified hygromycin resistance cassette, *hygSDM*. The functionality of *hygSDM* was confirmed by growth on hygromycin-supplemented LB agar. The erythromycin cassette from pSAM*erm* was then excised with the restriction enzymes MfeI and XbaI and replaced with the *hygSDM* resistance cassette from pCR®2.1-TOPO+*hygSDM,* which had the homologous ends added previously with KEB306 and 307. The plasmid was fully sequenced and transformed into *E. coli* β3914.

Creation of transposon mutant libraries

**DAY 1 & 2**

1. Streak out donor β3914(pSAM*hygSDM*) with recipient *K. pneumoniae* strains on LB agar supplemented with carbenicillin and DAP
2. Inoculate 5 mL overnight cultures of strains (Day 2)
   - β3914(pSAM*hygSDM*) in LB with carbenicillin and DAP at 37C ON
   - *K. pneumoniae*

**DAY 3**

1. Centrifuge 1.5 mL donor strain and 750 μl recipient strain for 1min to pellet bacteria.
2. Remove supernatant and resuspend pellet in 450 ul LB.
3. Pipet 10 μl mating spots onto LB plates.
4. Mate 1 hr at 37^o^C.
5. After mate, use a loop to resuspend spots in 14.5 mL LB. Be careful not to scrape up agar, which makes homogenizing difficult. Add more LB if too thick.
6. Homogenize well with a pipetman. Do not vortex.
7. Add equal volume of 50% glycerol.
8. Aliquot 2 mL into cryotubes and store at -80C.

**Day 4**

1. Thaw an aliquot on ice and plate 125 μl onto 15 cm LB agar plates with hygromycin.
2. Save 50 μl to serially dilute and drip plate for quantification.
3. Incubate all plates at 37^o^C ON.

**Day 5**

1. Count colonies on the drip plates. Can also count a quarter section of the large plates to estimate number of total colonies.
2. Use a cell scraper to resuspend colonies in 15 mL of LB. Add more if the solution is too thick.
3. Add an equal volume of 50% glycerol.
4. Divide majority of volume in 1 mL aliquots. Create a set of 100 μl aliquots with the remainder.

**Note**: The amount of mating spots you will need to reach a target number of CFU of mutants (e.g., 150,000) can be calculated by performing pilot experiments to determine mating efficiency. Additionally, a small aliquot of the frozen resuspended mate can be first thawed, serially diluted, and plated on LB agar supplemented with hygromycin to determine if the volume you have will produce enough CFU. For our libraries, we spread on about 110 15-cm-diameter plates to get enough separation between colonies.

Arbitrary PCR for transposon insertion sites in *K. pneumoniae*

**Primers**

Round 1

pSAMseq1 (Transposon-specific primer): TGCGAGAGTAGGGAACTGCCAGG

Arbitrary primers components: sequence introduced for Round 2 – random nucleotides – sequence that occurs throughout genome

Arb1: GGCCACGCGTCGACTAGTAC-N_10_-GATAT

Arb5: GGCCACGCGTCGACTAGTAC-N_10_-CAAGG

Arb6: GGCCACGCGTCGACTAGTAC-N_10_-ACGCC

Arb9: GGCCACGCGTCGACTAGTAC-N_10_-CGACG

**Note:** Arb5 and Arb6 give bands for most of the mutants. You can start with those two and then perform the PCR with Arb1 or Arb9 for any that do not produce a unique band from Arb5 and Arb6.

Round 2

pSAMseq2 (Transposon-specific primer): CTGTTGTTTGTCGGTGAACGCTCTC

Arb2: GGCCACGCGTCGACTAGTAC

**Arbitrary PCR Round 1**

| 9.75 μl | Water |
| --- | --- |
| 1 μl | DNA |
| 1.25 μl | Arb(1,5,6, or 9) |
| 0.5 μl | pSAMseq1 |
| 12.5 μl | GoTaq Green Master Mix (Promega) |
| 25 μl |  |

**Arbitrary PCR Round 1 Cycling Conditions**

94^o^C x 3 min

*94^o^C x 45 sec

30^o^C x 30 sec

72^o^C x 90 sec

**Repeat 6x from *

*94^o^C x 45 sec

45^o^C x 30 sec

72^o^C x 120 sec

**Repeat 30x from *

72^o^C x 10 min

**Arbitrary PCR Round 2**

| 9 μl | Water |
| --- | --- |
| 2.5 μl | Product from Round 1 |
| 0.5 μl | Arb2 |
| 0.5 μl | pSAMseq2 |
| 12.5 μl | GoTaq Green Master Mix |
| 25 μl |  |

**Cycling Conditions**

94^o^C x 3 min

*94^o^C x 45 sec

55^o^C x 40 sec

72^o^C x 90 sec

**Repeat 30x from *

72^o^C x 10 min

**Gel**

Run on 1.5% (w/v) agarose gel.

Extract unique bands for Sanger sequencing with pSAMseq2

Transposon Insertion Sequencing Data Analysis

Reads were first processed using Bowtie^7^ to trim barcodes and transposon sequences and to align the results to a reference genome^8^. Reads initially lacking a transposon sequence and reads aligning to the last 10% of a gene’s sequence were discarded. To identify colonization factors, ESSENTIALS compiled the ratio of the insertion site read numbers for each gene in the output pools to the insertion site read numbers in the input pool. Genes enriched and depleted in the output pools relative to the input pools were identified using a threshold of -2 or +2 logFC, respectively, and an FDR >0.05.

To identify genes that were necessary for growth in LB (referred to as “essential”), ESSENTIALS simulated a “perfect experiment” in which every TA site (the dinucleotide recognized by the Himar1 mariner transposon) received a transposon insertion and generated the same number of reads. Then, the logFC was calculated between these simulated number of reads per gene and those that were actually recovered from sequencing of the input pools. On the resulting density plot of logFC vs. number of genes, two peaks were observed, reflecting genes with very few reads because of limited growth and genes with numerous reads because of abundant growth. The local minimum between the two peaks was calculated and designated as the threshold for considering a gene “essential” for growth in LB. These genes were removed from the analysis of the output pools from the colonization experiments. One limitation of the INSeq approach and use of ESSENTIALS is that sequencing reads aligning to repetitive sequences in the genome cannot be unambiguously assigned to a particular gene and are discarded. Thus, repetitive regions may be automatically sorted as “essential genes” since they have no assigned reads. For this reason, we removed all genes categorized as “essential” if they had multiple copies in the genome.

Isogenic Mutant Construction

All isogenic deletion mutants in this study were created with the following method to replace the loci with an apramycin-resistance cassette. The 1000 bp regions upstream and downstream of the target loci for deletion were amplified by PCR with the primers listed in Supplemental Table 3. These primers were designed to contain overlap sequences with primers used to amplify the apramycin-resistance cassette from pIJ773^5^. These fragments were ligated together and into the EcoRI site of pUC18R6K with Gibson assembly (New England BioLabs). The resulting plasmids was propagated in *E. coli* PIR1.

Next, Lambda red machinery was introduced into *K. pneumoniae* strains by electroporation of pACBSR, which was maintained by growth at 30^o^C and supplementation with hygromycin. The lambda red machinery was induced by growing the *K. pneumoniae* strain in 5 mL LB supplemented with hygromycin and 475 μl of 1 M arabinose for 3.5 hours at 30^o^C with shaking. Then, *K. pneumoniae* bacteria were made competent and electroporated with a DNA fragment containing an apramycin-resistance cassette flanked by upstream and downstream regions of the gene of interest, which had previously been amplified by PCR from the pUC18R6K construct described above.

the fragment described above. Cells were recovered in LB for 2.5 hours at 37^o^C with shaking. Mutants were verified by Sanger sequencing, and pACBSR was removed by inoculating bacteria into LB, growing them overnight at 37^o^C without selection, plating for single colonies, and screening for plasmid loss by patching onto LB agar supplemented with apramycin or hygromycin.

To create marked parental strains, homologous overhangs were amplified for the Tn7 insertion site in each strain using primers listed in Supplemental Table 3. These were ligated using Gibson cloning to amplified apramycin- or hygromycin-resistance cassettes, as described above. For insertion of the hygromycin cassette, a modified version of pACBSR was necessary because pACBSR also contains HygR. The original HygR in pACBSR was replaced with an apramycin-resistance cassette as follows. pACBSR was digested with XhoI and BglII to excise HygR. Primers AprR_F and AprR_R were then used to amplify the apramycin cassette from pIJ773 and add overlap sequences designed to hybridize to the ends of the digested pACBSR plasmid. After ligation with Gibson assembly, the resulting plasmid was named pACBSR*apr*. Transformation into *K. pneumoniae* and curing of pACBSR or pACBSR*apr* was achieved as described above.

For the creation of the Δ*acrA* complemented strain in CRE-166, designated CRE-166Δ*acrA* Tn7::*acrA*, we took advantage of FRT sites flanking the apramycin-resistance cassette within the Δ*acrA* allele. We transformed CRE-166Δ*acrA*::AprR with pFLP-hyg to excise this cassette. Hygromycin-resistant colonies were cultured in LB at 37^o^C with shaking overnight and plated for single colonies. The colonies were screened for excision of the apramycin-resistance cassette and curing of pFLP-hyg by patching onto LB agar with or without hygromycin or apramycin. After the apramycin-resistance cassette was flipped out and pFLP-hyg was cured, the resulting strain (designated CRE-166Δ*acrA*) was transformed with pACBSR. Then the *acrA* gene along with 142 nucleotides of upstream DNA were PCR amplified from CRE-166 and ligated with a downstream apramycin-resistance cassette between homologous overhangs to the Tn7 site as above. This fragment was transformed into electrocompetent CRE-166Δ*acrA*, and the screening and pACBSR curing process was completed as described above. The Δ*tatABCD*-complemented strain in CRE-166 was created in the same fashion.

References

1. Bulman ZP, Krapp F, Pincus NB, et al. Genomic Features Associated with the Degree of Phenotypic Resistance to Carbapenems in Carbapenem-Resistant *Klebsiella pneumoniae*. mSystems 2021;6.

2. Kochan TJ, Nozick SH, Medernach RL, et al. Genomic surveillance for multidrug-resistant or hypervirulent *Klebsiella pneumoniae* among United States bloodstream isolates. BMC Infectious Diseases 2022;22.

3. Bolger AM, Lohse M, Usadel B. Trimmomatic: a flexible trimmer for Illumina sequence data. Bioinformatics 2014;30:2114.

4. Wick RR, Judd LM, Holt KE. Performance of neural network basecalling tools for Oxford Nanopore sequencing. Genome Biology 2019;20.

5. Huang T-W, Lam I, Chang H-Y, Tsai S-F, Palsson BO, Charusanti P. Capsule deletion via a λ-Red knockout system perturbs biofilm formation and fimbriae expression in *Klebsiella pneumoniae* MGH 78578. BMC Research Notes 2014;7:13.

6. Goodman AL, Wu M, Gordon JI. Identifying microbial fitness determinants by insertion sequencing using genome-wide transposon mutant libraries. Nature Protocols 2011;6:1969–80.

7. Langmead B, Trapnell C, Pop M, Salzberg SL. Ultrafast and memory-efficient alignment of short DNA sequences to the human genome. Genome Biology 2009;10:1–10.

8. Wang N, Ozer EA. A Method for Bioinformatic Analysis of Transposon Insertion Sequencing (INSeq) Results for Identification of Microbial Fitness Determinants. Methods in Molecular Biology (Clifton, NJ) 2017;1498:;.
